# A High Andean Hydrocolloid Extracted by Microatomization: Preliminary Optimization in Aqueous Stability

**DOI:** 10.3390/polym16131777

**Published:** 2024-06-23

**Authors:** Yudith Choque-Quispe, David Choque-Quispe, Carlos A. Ligarda-Samanez, Aydeé M. Solano-Reynoso, Sandro Froehner, Betsy S. Ramos-Pacheco, Yakov Felipe Carhuarupay-Molleda, Liliana Asunción Sumarriva-Bustinza

**Affiliations:** 1 Department of Environmental Engineering, Universidad Nacional José María Arguedas, Andahuaylas 03701, Peru; 2 Water and Food Treatment Materials Research Laboratory, Universidad Nacional José María Arguedas, Andahuaylas 03701, Peru; bsramos@unajma.edu.pe; 3 Research Group in the Development of Advanced Materials for Water and Food Treatment, Universidad Nacional José María Arguedas, Andahuaylas 03701, Peru; caligarda@unajma.edu.pe; 4 Nutraceuticals and Biopolymers Research Group, Universidad Nacional José María Arguedas, Andahuaylas 03701, Peru; 5 Department of Agroindustrial Engineering, Universidad Nacional José María Arguedas, Andahuaylas 03701, Peru; 6 Food Nanotechnology Research Laboratory, Universidad Nacional José María Arguedas, Andahuaylas 03701, Peru; 7 Department of Basic Sciences, Universidad Nacional José María Arguedas, Andahuaylas 03701, Peru; amsolano@unajma.edu.pe (A.M.S.-R.); ycarhuarupay@unajma.edu.pe (Y.F.C.-M.); 8 Department of Environmental Engineering, Federal University of Parana, Curitiba 80010, Brazil; froehner@ufpr.br; 9 Academic Department of Chemistry, Faculty of Science, Universidad Nacional de Educación Enrique Guzman y Valle, Lurigancho-Chosica 15472, Peru; lsumarriva@une.edu.pe

**Keywords:** *Nostoc sphaericum*, sedimentation, ζ potential, color index

## Abstract

Aqueous suspensions rely on electrostatic interactions among suspended solids, posing a significant challenge to maintaining stability during storage, particularly in the food and pharmaceutical industries, where synthetic stabilizers are commonly employed. However, there is a growing interest in exploring new materials derived from natural and environmentally friendly sources. This study aimed to optimize the stability parameters of a novel Altoandino Nostoc Sphaericum hydrocolloid (NSH) extracted via micro atomization. Suspensions were prepared by varying the pH, gelatinization temperature and NSH dosage using a 2^3^ factorial arrangement, resulting in eight treatments stored under non-controlled conditions for 20 days. Stability was assessed through turbidity, sedimentation (as sediment transmittance), ζ potential, particle size, color and UV-Vis scanning. Optimization of parameters was conducted using empirical equations, with evaluation based on the correlation coefficient (*R*^2^), average relative error (*ARE*) and *X*^2^. The suspensions exhibited high stability throughout the storage period, with optimized control parameters identified at a pH of 4.5, gelatinization temperature of 84.55 °C and NSH dosage of 0.08 g/L. Simulated values included turbidity (99.00%), sedimentation (72.34%), ζ potential (−25.64 mV), particle size (300.00 nm) and color index (−2.00), with simulated results aligning with practical application. These findings suggest the potential use of NSH as a substitute for commercial hydrocolloids, albeit with consideration for color limitations that require further investigation.

## 1. Introduction

The food, polymer and pharmaceutical industries are increasingly prioritizing the utilization of environmentally friendly plant materials to foster a circular and sustainable economy [1,2]. This shift has led to a significant focus on harnessing the potential of marine algae, seeds, plants and fruits in the production of hydrocolloids [3,4,5]. Notably, freshwater algae emerge as a promising alternative, offering a rich source of hydrocolloids capable of replacing traditional stabilizers while delivering comparable or even superior qualities. This is attributed to their high content of polysaccharides (such as pectins, carrageenans, alginates and gums) as well as proteins with exceptional gelling properties [6,7,8,9].

Hydrocolloids are characterized as highly hydrophilic substances of considerable molecular weight, exhibiting excellent solubility and hydration properties in water [10,11]. Predominantly sourced from biological origin such as algae, seeds and tubers, they are primarily composed of polysaccharides [12,13,14]. These polysaccharides possess diverse capabilities to form gels or act as thickeners across various conditions and even at low concentrations, thereby forming colloids with particular structures [5,15,16].

Moreover, their versatile functional properties as thickeners, gelling agents and stabilizers in suspensions enable the modification and conditioning the rheological behavior of aqueous systems [17,18]. These properties facilitate the capture and retention of moisture, the inhibition of liquid evaporation, the stabilization of foams and emulsions and control over rheology, freezing rate and ice crystal formation [19]. However, it is essential to note that these qualities are contingent upon the source of origin and have been extensively researched [7,12,20].

Some of the most commercialized hydrocolloids include guar gum, xanthan gum, sodium alginate, pectins, carrageenan (including i-carrageenan and k-carrageenan), gelatin and locust bean gum, among others. These highly functional compounds have sparked revolutions in the food, pharmaceutical and water treatment industries [21,22,23]. Hydrocolloids are available in the market, categorized by their synthetic, semi-synthetic and natural origin [12,24,25,26]. However, in light of increasing consumer consciousness regarding environmental preservation and sustainability, natural hydrocolloids are increasingly preferred [27].

Commercial hydrocolloids are typically extracted through processes utilizing strong or weak acids and alkalis, often incorporating salts [22,23,27,28]. These extraction methods commonly involve stages of size reduction and water elimination. However, employing gentle extraction techniques could help preserve their properties. For instance, spray drying with concentrated or slightly diluted juices presents a promising avenue for achieving this goal.

Although marine algae have been extensively studied and utilized in the food industry, scientific knowledge regarding freshwater algae, particularly their extracted derivatives such as hydrocolloids, remains limited. One such example is *Nostoc sphaericum*, a renewable natural resource thriving in aquatic environments in the Andes of Peru at altitudes exceeding 3800 m [7,29,30,31,32]. Traditionally, it has been consumed as food by high Andean inhabitants, often incorporated into stews for its thickening properties or enjoyed in salads.

The stability of hydrocolloids in aqueous environments is contingent upon various factors, including hydrocolloid concentration, pH, temperature and mixing speed. This stability can be elucidated by considering parameters such as ζ potential, particle size, molecular weight, shear stress, deformation rate and activation energy [32,33,34,35]. Commercial hydrocolloids are characterized by these control parameters, and thus, determining them is crucial for assessing the potentiality of a new hydrocolloid.

Conversely, the demand for hydrocolloids hinges upon their inherent qualities and benefits, with a crucial aspect being the optimal dosage and its impact on the properties of the solvent medium. This includes considerations such as the stability of suspended solids, color preservation, viscosity, rheological behavior, gel formation, the control of release and retention of active ingredients, sensory attributes, compatibility with other constituents and the modification of functional properties during storage, which is especially pertinent when utilized for edible or pharmaceutical purposes [8,32,36,37,38,39,40,41]. Consequently, it is imperative to ascertain the precise conditions under which a new hydrocolloid exhibits these characteristics in an aqueous medium and with varying doses of NSH (*Nostoc sphaericum* hydrocolloid), pH levels and processing temperatures.

A pivotal consideration in the application of stabilizers is their ability to maintain particle suspensions in aqueous mixtures over extended storage durations. Consequently, hydrocolloids derived through the microatomization of *Nostoc sphaericum* may offer this advantage. Nonetheless, it is imperative to establish the optimal conditions under which this hydrocolloid exhibits its greatest efficacy.

The utilization of empirical mathematical models enables the optimization process, facilitating the determination of precise conditions and parameters for studying hydrocolloid stability. This approach ensures that its usage can be reliably guaranteed under specific processing and application conditions.

Hence, this research aimed at optimizing parameters influencing the stability of the hydrocolloid extracted via microatomization from Nostoc Sphaericum. This optimization involved assessing key factors such as turbidity, color, sedimentation, ζ potential and particle size within aqueous suspensions.

## 2. Materials and Methods

### 2.1. Raw Material

The *Nostoc sphaericum* hydrocolloid (NSH) was provided by the Laboratory of Materials Research for Water and Food Treatment of the Universidad Nacional José María Arguedas, Peru. Wild algae specimens were harvested from the wetlands of the José María Arguedas district, Andahuaylas, Peru, located at coordinates 13°47′52″ S, 73°17′55″ W at an altitude of 4251 m. Following collection, the samples underwent thorough washing with abundant distilled water. Subsequently, they were blended in distilled water at a ratio of 1/1 (*w*/*w*) and sieved to a particle size of 45 µ. The resulting algae suspension underwent spray drying using a Mini Spray Dryer B-290, Büchi Labortechnik AG, Flawil, 106 Switzerland. The drying parameters were set as follows: an inlet temperature of 120 °C, gas spraying at 650 L/h, an aspiration rate of 85%, a feed rate of 5 mL/min and a two-fluid nozzle with an internal diameter of 0.7 mm.

### 2.2. Suspension Conditioning

Suspensions were meticulously prepared as outlined in Table 1, combining NSH with chlorine-free drinking water characterized by specific parameters including total dissolved solids (262 mg/L), hardness (255 mg/L), conductivity (520 μS/cm), color (0.0), pH (7.67), fluorides (0.29 mg/L) and turbidity (0.0 NTU). The pH of the suspension was carefully adjusted using citric acid (SpectrumChemical Mfg. Corp., Bathurst, NB, Canada). To prevent microbial proliferation, potassium sorbate obtained from Scharlau, Sentmenat, Spain, was introduced at a concentration of 10 ppm. Subsequently, the suspensions were heated to both 60 and 80 °C, representing the minimum and maximum viscosity range, respectively [32], while being continuously stirred at 60 rpm using a model M6 CAT from Germany. Finally, the suspensions were transferred to 1.6 cm diameter glass test tubes and securely sealed for storage under ambient conditions for a duration of 20 days.

### 2.3. Suspension Stability Evaluation

#### 2.3.1. Rheological Measurements

The rheological tests were carried out using an Anton Paar rheometer, specifically the model MCR702e (Graz, Austria), equipped with Peltier units for precise thermal control. The apparent viscosity behavior and shear rate of the suspensions were determined over a shear rate range of 1 to 300 s^−1^ at 60 and 80 °C, employing a concentric cylinder geometry for each treatment [42,43]. The consistency index *k* in units of Pa.s^n^ and the behavior index (*n*) were determined utilizing the Power Law for non-Newtonian fluids (Equation (1)).

(1) $$\tau =k\gamma^{n},$$

Constants *k* and *n* were determined by nonlinear regression using the least difference of squares through the Quasi Newton method [44].

#### 2.3.2. Turbidity Measurements

Additionally, turbidity was assessed by transferring 20 mL of each treatment into 1.6 cm diameter test tubes. Following a 20-day storage period, 10 mL was extracted from the top layer, and its absorbance was measured at 560 nm using a UV-Vis spectrophotometer (Genesys 150 UV, Thermo Fisher, Waltham, MA, USA). To evaluate sedimentation, 8 mL of the remaining 10 mL was discarded, leaving behind 2 mL that was vigorously shaken at 3000 rpm for 2 min using a vortex mixer (model 2VH, Boeco, Hamburg, Germany), followed by absorbance measurement.

#### 2.3.3. Particle Size and ζ Potential Measurements

Particle size, represented by the average diameter, was determined as follows: 2 mL of the sample underwent sonication for 3 min using the Sonics VCX 750 instrument (Newtown, CT, USA), followed by an analysis with dynamic light scattering (–DLS) equipment, specifically the Zetasizer ZSU3100 (Malvern Instruments, Worcestershire, UK), operating at a laser wavelength of 632.8 nm, a scattering angle of −14.14° and an electric field strength of 5 V/cm. An aliquot of the sample was injected into a DTS1070 capillary cell to determine the ζ potential.

The final results of the rheological parameters, turbidity, sedimentation, particle size and ζ potential were obtained from Choque-Quispe et al. [32].

#### 2.3.4. Color Measurements

The color stability of the suspensions was assessed within the CIE L* a* b* color space, employing specific criteria. Luminosity (L*) was measured on a scale ranging from 0 = black to 100 = white, and chroma values a* and b* were utilized to characterize color properties (+a = red, −a = green, +b = yellow and −b = blue) [45]. For this analysis, treatment samples were examined using a Konica Minolta colorimeter, model CR-5 (Tokyo, Japan), with readings recorded in the reflectance module. Furthermore, the color index (CI*), yellow index (*YI*) and green index (*GI*) were calculated using Equations (2)–(4), respectively, providing a quantitative representation of the color [46]:

− If the CI* is −40 to −20, colors range from blue-violet to deep green.
− If the CI* is −20 to −2, colors range from deep green to yellowish green.
− If the CI* is −2 to +2, this represents greenish yellow.
− If the CI* is +2 to +20, colors range from pale yellow to deep orange.
− If the CI* is +20 to +40, colors range from deep orange to deep red.

(2) $${CI}^{*}=\frac{a^{*}\cdot 1000}{L^{*}\cdot b^{*}},$$

(3) $$YI=\frac{\left(138.17\cdot L^{*}-129.99)b^{*}\right)}{L^{*}}$$

(4) $$GI=\frac{a^{*}}{{\left({a^{*}}^{2}+{b^{*}}^{2}\right)}^{1/2}}$$

### 2.4. UV-Vis Sscanning

The UV-Vis spectra absorbance of the suspensions was measured across the range of 200 to 800 nm with a step size of 2 nm, employing ultrapure water as a blank reference. This analysis was conducted using a UV-Vis spectrophotometer (Genesys 150 UV, Thermo Fisher, Waltham, MA, USA). The obtained spectra were plotted to illustrate notable alterations in the treatments resulting from variations in pH and NSH concentration.

### 2.5. Chemometric Analysis by PCA

Chemometric analysis of the UV-Vis spectra was conducted using Principal Component Analysis (PCA), enabling the identification of elements contributing to variability in a data matrix M, consisting of n objects defined by m variables [47,48]. In this study, the spectra of each NSH solution were treated as objects, with the variables representing wavelengths spanning from 200 to 800 nm. Origin Pro 2022 software tools were utilized to standardize and analyze the data. The outcomes facilitated the identification of variability factors, notably wavelengths exhibiting the greatest variability across treatments, corresponding to characteristic functional groups.

### 2.6. Suspension Stability Parameter Optimization

The experimental data from the eight treatments (T1 to T8) were fitted to linear models with interactions, as described by Equation (5).

(5) $$Y=\beta_{0}+\sum_{i=1}^{k}\beta_{i}x_{i}+\sum_{1=1}^{k-1}\sum_{j=2}^{k}\beta_{ij}x_{i}x_{j}+e_{i},$$

where *x_i_* and *x_j_* represent the independent variables (pH, NSH dose, temperature) and *β*_0_, *β_i_* and *β_ij_* are the intercept, linear and intercept coefficients, respectively. The variable *k* represents the number of variables.

The optimal levels of the independent variables were based on the response variables: sedimentation, turbidity, ζ potential, particle size and color index. These were collectively considered an objective function aimed at maximizing the transmittance of sedimentation, serving as an indication of a lower solids content in the sediment on day 20 of storage, a time point where slight variability in the studied parameters was observed.

The adequacy of the models was assessed utilizing statistical tools for error function analysis. This included calculating the coefficient of determination (*R*^2^*_DC_*), which measures variability explained by the adjusted model, and the adjustment coefficient (*R^2^_AC_*), which, in addition to variability, considers the number of variables in the model, penalizing irrelevant ones. Furthermore, the average relative error (*ARE*) was calculated to minimize the distribution of fractional errors, and the Chi-squared test (*X*^2^), based on the least difference of squares, was employed [49,50,51]. These metrics were determined using Equations (6) and (7), respectively.

(6) $$ARE=\frac{100}{N}\sum_{i=1}^{N}{\left|\frac{X_{adj}-X_{exp}}{X_{exp}}\right|}_{i},$$

(7) $$X^{2}=\sum_{i=1}^{N}{\left[\frac{{\left(X_{exp}-X_{adj}\right)}^{2}}{X_{adj}}\right]}_{i},$$

where *X_adj_*, is the value reported by the model; *X_exp_* is the experimental value; and *N* is the amount of data.

*R*^2^ values close to unity indicate a good model fit, and *ARE* and *X*^2^ should be as low as possible.

### 2.7. Statistical Analysis

The data were collected in triplicate, and Tukey’s multiple comparisons tests were conducted at a significance level of 5%. Data analysis was carried out using Excel spreadsheets and the Solver utility, as well as Statistica V12 software (demo mode) and Origin Pro 2022 software.

## 3. Results and Discussion

### 3.1. Suspended Solid Stability of NSH Suspensions

The rheological study was conducted using the Ostwald–de Waele model, also known as the Power Law. This model, characterized by two constants, *k* and *n*, correlates the dissolved solids to the polarity of the liquid medium, providing insights into the dynamics and stability of the suspension [43,52,53].

We observed that the NSH suspensions exhibited non-Newtonian behavior of the dilatant type, with a behavior index (*n*) greater than 1.00 (Table 2). This indicated that their apparent viscosity increased with the deformation speed, resulting in enhanced thickening, more stability and reduced dispersion [54]. Such behavior arises from molecular interactions among hydrocolloid particles when subject to external stresses [55]. This characteristic is desirable in suspensions, as it hinders the sedimentation of suspended solids and the agglomeration of dissolved solids. Furthermore, variations in suspension pH are correlated with shear stress, with higher pH levels leading to increased shear stresses [56]. Consequently, as pH rises, NSH particles agglomerate under shear, resulting in softer suspensions with higher viscosity at low shear rates [56,57].

The observed low values of the consistency index (*k*) suggest ease of homogenization, indicating favorable characteristics for the hydrocolloid´s application as a stabilizer in juices or nectars [58,59]. Notably, at a pH of 6.5, treatments T1, T2, T5 and T6 exhibited higher *k* and lower *n* values. This phenomenon can be attributed to the isoelectric point (approximately a pH of 8.0), promoting attractive interactions between the particles [32,60].

In contrast, the initial turbidity of NSH suspensions exhibited a range of 98.38 to 99.89%, with a marginal decline observed post storage, ranging from 97.57 to 99.77% (Table 2), indicative of effective retention of solids in suspension. Initially, settleable solids, which resided at the container´s bottom, ranged from 73.95 to 82.85%, demonstrating desirable high transmittance values. However, following a 20-day storage period, these values experienced a slight reduction to a range of 63.17% to 77.09%, suggesting the phenomenon of resuspension and hydration of NSH particles. Consequently, treatments featuring a lower pH and reduced doses of SDH exhibited heightened turbidity, although no discernible consistent trend in sedimentation was observed (Table 2).

Factors such as hydrocolloid topography, size and ionic strength play pivotal roles in determining the stability of suspensions, a parameter that can be effectively assessed through the measurement of both ζ potential and particle size [61,62]. The ζ potential, indicative of the electrostatic repulsion between adjacent particles [61], is influenced by the interaction of ionizable functional groups (-OH, -COO-, -NH_2_ and −SO_3_H) with water via hydrogen bonds and electrostatic forces [63,64]. The observed ζ potential values for NSH suspensions ranged from −29.26 to −22.95 mV (Table 3), with slight variations reported after storage, ranging between −26.41 and −23.62 mV. Notably, no significant alterations were observed for T4, T5 and T7, potentially attributed to pH-induced modifications in the electric charges of NSH functional groups.

These findings indicate robust stability in suspensions prepared with NSH. Regarding the particle size of the NSH in solution, initial measurements revealed notably high values ranging from 626.17 to 941.00 nm, which subsequently experienced a substantial reduction over the storage period, converging to values spanning from 316.33 to 473.13 nm (Table 3).

The reduction in particle size can be attributed to the disintegration of NSH facilitated by electrophoretic mobility, leading to a decrease in the ζ potential [65,66]. Moreover, larger particles tend to sediment, thereby enriching the suspension with smaller counterparts. Nonetheless, no discernible pattern of alteration was evident across treatments, which could be attributed to the complex composition of SDH, primarily comprising carbohydrates, fats and proteins [8].

### 3.2. Color Stability of NSH Suspensions

Color represents a critical sensory attribute in food products, playing a pivotal role in consumer purchase decisions. It is intricately tied to the specific hues imparted by the constituents comprising the product. However, color can undergo alterations during storage, particularly in the case of naturally derived pigments [67,68].

**Figure 1:**
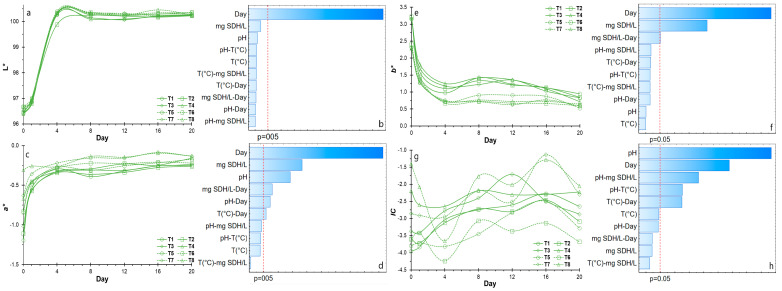
(**a**) Variation for L*; (**b**) Effects for L*; (**c**) Variation for a*; (**d**) Effects for a*; (**e**) Variation for b*; (**f**) Effects for b*; (**g**) Variation for CI*; (**h**) Effects for CI*.

The stability of hydrocolloid suspensions is closely linked to color indices such as the color index (CI*), yellow index (*YI*) and green index (*GI*), which serve as essential quality parameters for stored products and are indicative of turbidity and suspended solids [69,70,71]. Regarding the color stability of NSH suspensions, initial observations of the L* parameter ranged between 95.90 and 96.18, which notably increased to 99.86 after the 20-day storage period (Table 4), suggesting a progression toward lighter tones (Figure 1a,b). Conversely, chroma a* exhibited a tendency toward pale green, which stabilized on day 2 (Figure 1c), demonstrating the significant influence of storage duration, NSH concentration and pH (Figure 1d). Similarly, chroma b* displayed a trend toward pale yellow, stabilizing on day 4 (Figure 1e), with this parameter being notably impacted by storage duration and NSH concentration.

The color index (CI*) serves as a comprehensive metric for assessing the overall color of a food or material [32]. Throughout the storage period, observations revealed values spanning from −4.25 to −1.0 (Figure 1g), indicative of a prevailing global hue trending toward a pale greenish-yellow color. Notably, the stability exhibited a significant dependency on pH and storage duration (Figure 1h). Similarly, the yellow index (*YI*) reported values ranging between 311.7 and 436.0, reflecting hues characterized by very pale yellows (Table 4).

The slight color variation on day zero and during subsequent storage periods can be attributed to factors such as ambient light intensity [72]. Principal contributors to the coloration of plant-derived materials like NSH include chromophoric compounds such as chlorophyll, carotenoids, lycopene, betalains, phenols and phycobiliproteins [67,73,74,75], whose stability in natural and processed forms over time is lower compared to synthetic ones due to oxidative, enzymatic and Maillard reactions that occur in processed foods [76,77,78]. Therefore, although NSH presents promise as a natural additive in aqueous systems, owing to its notable color stability, it is imperative that such additives do not impart any additional coloration to the suspensions.

### 3.3. UV-Vis Scanning of NSH Suspensions

UV-Vis scanning represents a rapid, straightforward and non-destructive method suitable for samples in aqueous or liquid matrices. This technique enables the characterization and identification of compounds, discerning signals from both organic and inorganic constituents. Spectral information is typically observed in the visible (Vis) region from 400 to 800 nm, as well as in the ultraviolet (UV) range from 200 to 400 nm [79,80,81].

Conversely, UV-Vis analysis offers valuable insight into alterations in electronic energy levels within molecules resulting from electron transitions between distinct orbitals [82]. Emulsions and homogeneous suspensions, characterized by highly dispersed solutes, exhibit absorbance characteristics corresponding to various functional groups across different wavelengths, a phenomenon readily assessed through UV-Vis spectroscopy. However, the presence of heterogeneity within these systems can yield complex spectra, owing to diverse components including water, proteins, carbohydrates, lipids, proteins, vitamins and minerals [79,83].

**Figure 2:**
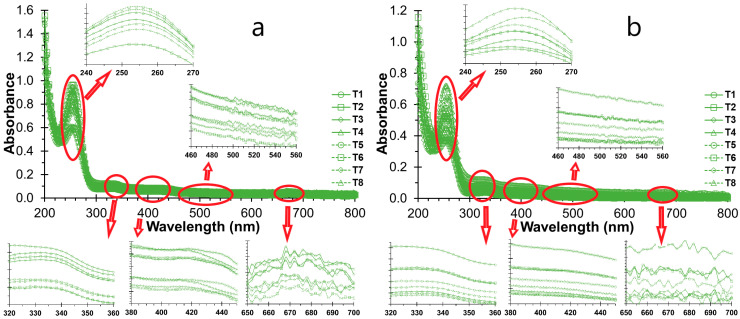
UV-Vis scan: (**a**) NSH suspension on day 0; (**b**) NSH solution on day 20.

The visible spectrum corresponds to the interval between 400 and 800 nm and encompasses the absorption bands of natural dyes and pigments [79,80]. For instance, chlorophylls and pheophytins exhibit an absorption peak between 650 and 700 nm [79,84], and anthocyanins typically absorb light between 460 and 560 nm [85,86]. NSH suspensions displayed a modest intensity on day 0 (Figure 2a), which markedly decreased by day 20 (Figure 2b). This phenomenon can be attributed to the agglomeration of hydrocolloids, leading to large particles that sediment and consequently entrap the pigments.

The UV region spans from 200 to 400 nm, where high-intensity spectra typically denote π–π* electronic transitions associated with compounds containing aromatic rings [82]. Carotenoid pigments, for instance, exhibit absorption peaks within the range of 380 to 450 nm [84]. Similarly, porphyrin rings found in chlorophyll a and b, carotenoids like fucoxanthin, polyphenols and antioxidant compounds, albeit in lesser amounts in algae, manifest absorbance in the UV range of 240 to 270. Flavonoids possessing conjugated rings display maximum adsorption between 320 and 360 nm for flavones and flavonols [86]. The intensity of these peaks is notably prominent on day 0 but undergoes significant attenuation by day 20 of storage (Figure 2). This decline could be attributed to both external effects during storage, such as light exposure, and internal processes like oxidation [72,76,77,87].

### 3.4. PCA Analysis of UV-Vis Spectra

The UV-Vis scanning spectra of NSH suspensions were subject to Principal Component Analysis (PCA) to elucidate alterations in the compositions of aqueous suspensions. It was observed that variability was captured by two principal components, explaining 98.3% and 85.2% of the variance for samples collected on day 0 and day 20, respectively.

**Figure 3:**
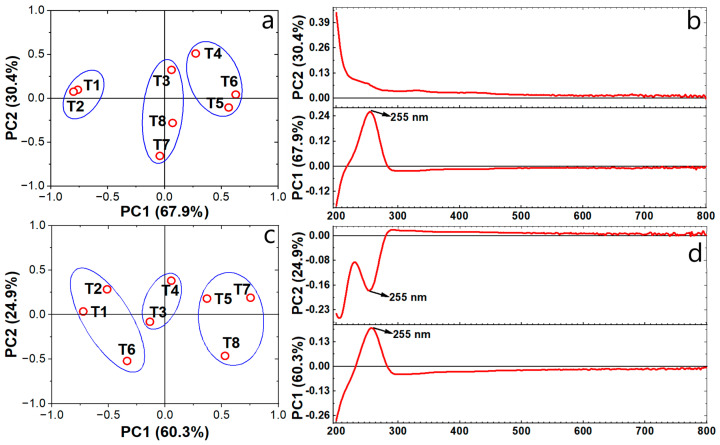
PCA for UV-Vis scanning for NSH solution treatments: (**a**) Score plot for day 0; (**b**) Loading plot for day 0; (**c**) Score plot for day 20; (**d**) Loading plot for day 20.

In Figure 3a, three distinct groups are delineated based on PC1 (accounting for 67.9% of the variance), with positive loadings observed for T4, T5 and T6 on the initial day. However, by day 20, only T5 retained significant positive loadings (Figure 3c), suggesting that the T5 treatment maintained relatively stable levels of pigments such as chlorophyll, carotenoids, polyphenols and antioxidant compounds during storage. Conversely, T1 and T2 exhibited negative loadings at both the beginning and end of storage, whereas T3 displayed no discernible modification.

Conversely, the loading plot of the NSH suspension spectra reveals notable changes primarily within the UV spectrum (between 200 to 400 nm) (Figure 3b,d). This indicates that alterations predominantly occur at the structural level of the constituent molecules. Interestingly, compounds detected within the visible spectrum remain largely unaffected by storage duration. UV spectra play a pivotal role in assessing compositional variation in NSH suspensions during storage, particularly around 255 nm, as identified by PC1 on the initial day and by PC1 (60.3%) and PC2 (24.9%) on day 20. The positive loading values for PC1 (60.3%) of the loading plot for day 20 (Figure 3d) indicate that the treatments within group 1 maintained or enhanced their pigmented constituents. Conversely, the negative loading values for PC2 (24.9%) of the loading plot (Figure 3d) at 255 nm suggest a reduction or loss of pigmented constituents, corresponding to treatments T6 and T8 in Figure 3c.

Despite these spectral changes, alterations in the UV spectrum were not visually perceptible, highlighting the need for color index analysis, which demonstrated no apparent variation during storage, a characteristic behavior often observed in materials of biological origin [83,88,89].

### 3.5. PCA Analysis of Suspension Properties

**Figure 4:**
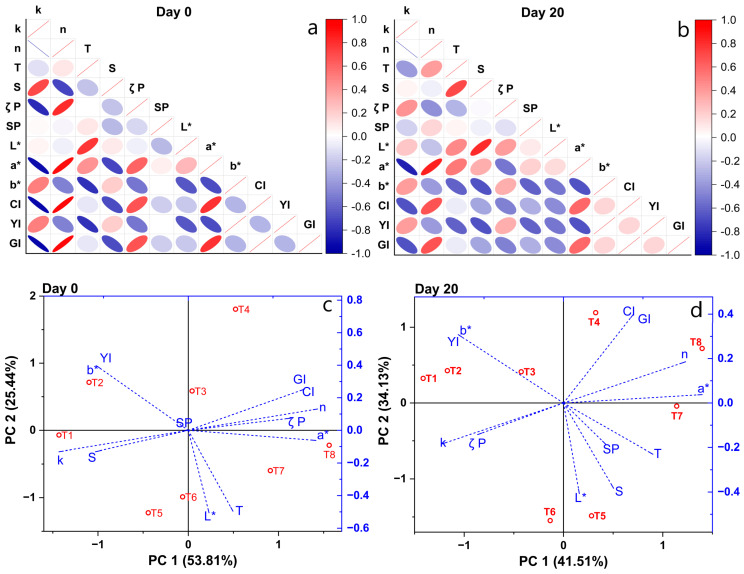
Correlation of stability parameters on (**a**) day 0 and (**b**) day 20. PCA of stability parameters on (**c**) day 0 and (**d**) day 20.

Several parameters influencing the stability of NSH suspensions exhibited both direct and inverse correlations initially, whereas by day 20, the number of independent parameters increased. Specifically, the ζ potential displayed an inverse correlation with k (consistency index) and a direct correlation with n (behavior index) initially, with the opposite trend observed by day 20 (Figure 4a,b). This phenomenon is likely attributed to the dispersion and hydration of the particles and molecules comprising NSH, leading to the formation of more stable electrical species. Similar trends were observed for turbidity and sedimentation.

In other instances, it was apparent that the dependence between parameters intensified over time, indicating significant stabilization of NSH during storage. This trend is evident in Figure 4c,d, where consistent grouping is observed for parameters such as CI*, *GI*, *n*, a*, T (turbidity) and L* in T4, T8 and T7. Similarly, treatments T2 and T1 exhibited consistent groupings for parameters including *YI*, b* and *k*.

### 3.6. NSH Suspension Parameter Optimization

Values of *R*^2^ > 0.7 serve as reliable indicators of a good fit when evaluating empirical models. However, in cases involving a large number of variables and treatments (in our scenario, *n* = 8), the complexity of the proposed models increases, allowing for greater variability, Therefore, it becomes imperative to consider additional statistics such as *ARE* and X^2^, where lower values are preferred, as they denote smaller discrepancies between experimental and adjusted data [49,50,51].

**Figure 5:**
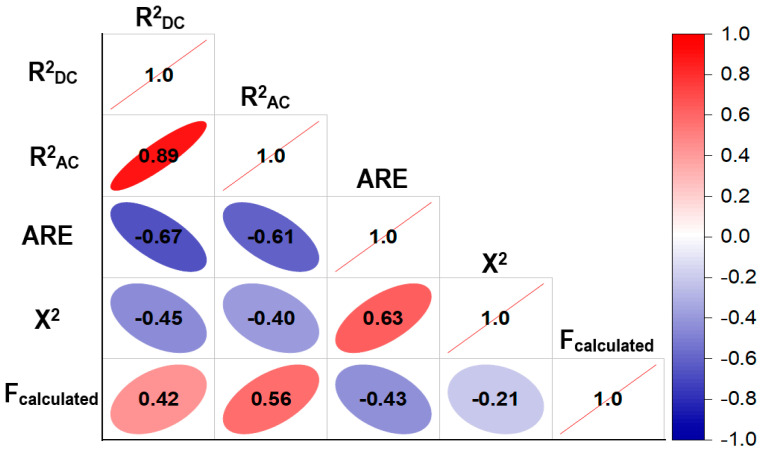
Correlation of model parameters.

The fitting of the experimental data revealed that the linear model with interactions exhibits significance (indicated by F_cal_ > F_crit_), alongside high values for *R*^2^_AC_ and *R*^2^_DC_ and low *ARE* and *X*^2^ values (Table 5). Consequently, this model demonstrates the capability to effectively model simulated data with high reliability. Additionally, the correlation among the statistics indicates a strong association between *ARE*, *X*^2^, *R*^2^_AC_ and *R*^2^_DC_ (Figure 5), thus justifying the decision to employ the linear model with interactions.

The adjusted models, as depicted by coefficients in Table 6, were used to optimize the independent variables, namely pH, temperature and NSH concentration. The objective function aimed to maximize sedimentation, expressed as the percentage of transmittance, resulting in optimal values of a pH of 4.50, a temperature of 84.55 °C and an NSH dose of 0.08 g/L (Table 7). The optimized properties of the suspension included 72.34% transmittance (sedimentation), 99.00% transmittance (turbidity), a −25.64 mV ζ potential, a 300.00 nm particle size in suspension and a −2.00 color index. These values collectively signify high stability within an aqueous medium for NSH.

The implementation of optimal parameters in an experimental trial yielded results closely resembling the optimal properties, including turbidity, ζ potential, sedimentation, particle size and color index. This consistency suggests that the NSH hydrocolloid holds significant promise as a versatile material for utilization in aqueous suspensions under diverse conditions.

## 4. Conclusions

The stability assessment of atomized Nostoc sphaericum hydrocolloid (NSH) demonstrated favorable stability over 20 days of storage under non-controlled conditions. It exhibited minor fluctuations in ζ potential and a reduction in particle size and displayed a pale yellow-greenish coloration, indicative of chromophores originating from the NSH, as assessed via UV-Vis spectroscopy. Suspension parameters were fitted to a linear model with interactions, yielding high *R*^2^_AC_ and *R*^2^_DC_ values alongside low *ARE* and *X*^2^ values. Optimal conditions were determined as a pH of 4.5, a gelatinization temperature of 84.55 °C and an NSH dosage 0.08 g/L for a 20-day storage period. Simulated values included 99.00% turbidity, 72.34% sedimentation, a ζ potential of −25.64 mV, a particle size of 300.00 nm and a color index of −2.00, exhibiting similar trends in practical application. These findings indicate the potential of NSH as a substitute for commercial hydrocolloids, albeit with considerations for color limitations that require further investigation.

## Figures and Tables

**Table 1:** Experimental design matrix.

Treatment	pH	Dose NSH (g/L)	Temperature (°C)
T1	6.5	0.10	60
T2	6.5	0.10	80
T3	4.5	0.10	60
T4	4.5	0.10	80
T5	6.5	0.07	60
T6	6.5	0.07	80
T7	4.5	0.07	60
T8	4.5	0.07	80

**Table 2:** Turbidity and sedimentation of NSH suspensions.

	*k* ***	*n* ***	Turbidity *** (% Transmittance)							Sedimentation *** (% Transmittance)						
			Day 0			Day 20			**	Day 0			Day 20			**
			$\bar{x}$	±s	*	$\bar{x}$	±s	*		$\bar{x}$	±s	*	$\bar{x}$	±s	*	
T1	2.5	1.38	98.71	0.01	a	97.57	0.52	c	<	77.49	0.22	b	72.61	0.5	a	<
T2	1.79	1.41	98.47	0.02	b	97.60	0.06	c	<	77.1	0.03	b	71.57	0.5	b	<
T3	1.12	1.52	98.64	0.06	c	97.90	0.1	c	<	74.81	0.16	c	63.17	0.29	c	<
T4	0.79	1.57	98.38	0.03	d	97.48	0.06	c	<	73.95	0.81	d	66.28	0.14	d	<
T5	1.37	1.45	99.42	0.01	e	99.06	0.49	b	<	82.85	0.40	a	77.09	0.22	e	<
T6	1.13	1.51	99.89	0.01	f	99.67	0.1	a	<	74.18	0.45	c,d	78.59	0.36	f	<
T7	0.97	1.55	99.63	0.03	g	99.33	0.11	a,b	<	72.95	0.62	e	73.73	0.22	g	<
T8	0.64	1.62	99.51	0.05	h	99.77	0.06	a	<	74.67	0.04	c,d	74.52	0.1	h	<

Ti is the treatment i; $\bar{x}$, arithmetic mean; s, standard deviation. * Evaluated through Tukey’s multiple comparisons test at 5% significance. ** Evaluated through Student’s *t*-test at 5% significance for paired samples. *n* = 5. *** Data taken from Choque-Quispe et al. [32].

**Table 3:** ζ potential and particle size in NSH suspensions.

	ζ Potential (mV)							Particle Size (nm)						
	Day 0			Day 20			**	Day 0			Day 20			**
	$\bar{x}$	±s	*	$\bar{x}$	±s	*		$\bar{x}$	±s	*	$\bar{x}$	±s	*	
T1	−27.35	1.87	b,c	−24.27	0.26	a	>	941.00	19.16	a	316.33	11.07	b	<
T2	−29.26	1.47	c	−25.22	1.45	a	<	626.17	33.04	d	342.17	27.94	b	<
T3	−23.18	1.29	a	−24.77	1.29	a	>	630.67	19.11	d	464.00	45.42	a	<
T4	−24.39	1.51	a,b	−24.98	0.79	a	>	884.03	53.28	a,b	363.03	34.15	b	<
T5	−25.87	1.62	a,b,c	−25.19	1.09	a	>	708.37	66.71	c,d	473.13	23.06	a	<
T6	−26.76	0.88	a,b,c	−23.62	0.31	a	<	725.67	88.15	c,d	344.37	26.33	b	<
T7	−26.08	0.72	a,b,c	−26.41	0.69	a	>	776.33	50.80	b,c	453.33	44.21	a	<
T8	−22.95	1.38	a	−26.20	1.28	a	>	688.97	32.12	c,d	309.97	8.07	b	<

Ti is the treatment i; $\bar{x}$, arithmetic mean; s, standard deviation. * Evaluated through Tukey’s multiple comparisons test at 5% significance. ** Evaluated through Student’s *t*-test at 5% significance for paired samples. *n* = 3.

**Table 4:** Color stability in NSH suspensions.

	L*							a*							b*						
	Day 0			Day 20			**	Day 0			Day 20			**	Day 0			Day 20			**
	$\bar{x}$	±s	*	$\bar{x}$	±s	*		$\bar{x}$	±s	*	$\bar{x}$	±s	*		$\bar{x}$	±s	*	$\bar{x}$	±s	*	
T1	95.92	0.01	f	99.79	0.01	b	<	−1.20	0.01	a	−0.25	0.01	d,e	<	3.16	0.01	c	0.95	0.01	a	<
T2	95.95	0.01	e	99.73	0.02	c	<	−1.11	0.01	b	−0.26	0.01	e	<	3.22	0.01	a	0.85	0.01	b	<
T3	95.93	0.01	f	99.74	0.01	c	<	−1.02	0.01	c	−0.24	0.01	c,d	<	3.15	0.01	c	0.83	0.02	b	<
T4	95.90	0.01	g	99.74	0.02	c	<	−0.67	0.01	d	−0.16	0.01	b	<	3.19	0.01	b	0.71	0.02	c	<
T5	96.18	0.01	a	99.85	0.02	a	<	−0.85	0.01	e	−0.17	0.01	b	<	2.32	0.01	e	0.52	0.01	f	<
T6	96.17	0.01	b	99.86	0.07	a	<	−0.82	0.01	f	−0.22	0.01	c	<	2.39	0.01	d	0.60	0.02	e	<
T7	96.14	0.01	c	99.77	0.02	b,c	<	−0.63	0.01	g	−0.13	0.01	a	<	2.31	0.01	e	0.58	0.02	e	<
T8	96.07	0.01	d	99.76	0.01	b,c	<	−0.31	0.01	h	−0.13	0.00	a	<	2.28	0.01	f	0.64	0.01	d	<
	CI*							***YI***							***GI***						
T1	−3.95	0.01	g	−2.65	0.13	b	<	431.9	0.71	c	129.57	0.71	a	<	−0.35	0.00	g	−0.26	0.01	b	<
T2	−3.60	0.03	e	−3.09	0.14	c,d	<	440.1	0.71	a	115.65	1.44	b	<	−0.33	0.00	e	−0.29	0.01	c,d	<
T3	−3.38	0.02	d	−2.88	0.06	b,c	<	431.4	0.71	c	112.92	2.84	b	<	−0.31	0.00	d	−0.28	0.01	b,c	<
T4	−2.18	0.03	b	−2.22	0.16	a	>	436.0	0.71	b	97.63	3.31	c	<	−0.20	0.00	b	−0.22	0.02	a	>
T5	−3.80	0.03	f	−3.28	0.22	d	<	317.0	0.71	e	71.63	1.66	f	<	−0.34	0.00	f	−0.31	0.02	d	<
T6	−3.58	0.04	e	−3.62	0.23	e	>	327.5	0.71	d	81.44	2.84	e	<	−0.33	0.00	e	−0.34	0.03	e	>
T7	−2.85	0.03	c	−2.30	0.16	a	<	315.8	1.35	e	79.84	3.08	e	<	−0.26	0.00	c	−0.22	0.02	a	<
T8	−1.40	0.02	a	−2.05	0.07	a	<	311.7	1.82	f	88.05	1.12	d	<	−0.13	0.00	a	−0.2	0.01	a	<

CI* is the color index; *YI* is the yellow index; *GI* is the green index; Ti is the treatment i; $\bar{x}$, arithmetic mean; s, standard deviation. * Evaluated through Tukey’s multiple comparisons test at 5% significance. ** Evaluated through Student’s *t*-test at 5% significance for paired samples. *n* = 5.

**Table 5:** Empirical model statistics.

NSH Suspension Property	Model	*R* ^2^_DC_	*R* ^2^_AC_	*ARE*	X^2^	F_calculated_	F_critical_
Sedimentation (%T)	L	0.9416	0.8978	1.3666	0.1580	21.5011	0.0063
	LITF	0.9844	0.8907	0.8467	0.0412	10.5095	0.2318
Turbidity (%T)	L	0.9545	0.9204	0.1818	0.0032	27.9894	0.0038
	LITF	0.9986	0.9902	0.0355	0.0001	119.0552	0.0700
Z potential (mV)	L	0.4559	0.0479	2.1507	0.1302	1.1173	0.4409
	LITF	0.9078	0.3545	1.0444	0.0219	1.6408	0.5353
Particle size (nm)	L	0.5551	0.2215	9.3394	35.8759	1.6638	0.3104
	LITF	0.9409	0.5864	4.0750	4.9762	2.6539	0.4381
Color index	L	0.5969	0.2947	9.8341	0.3408	1.9748	0.2599
	LITF	0.9889	0.9225	2.0976	0.0094	14.8829	0.1959

L, linear; LITF, linear with the interaction of two factors; *R*^2^_DC_, determination coefficient; *R*^2^_AC_, adjusted coefficient; *ARE*, average relative error; *X*^2^, Chi-squared test. Data were evaluated at 5% significance.

**Table 6:** Coefficients of the fitted models.

Coefficient	T	S	ζP	PS	CI
Intercept	100.04	85.37	−55.30	1570.49	0.28
pH	−0.73	0.62	2.47	20.17	−0.29
T	0.07	0.31	0.17	−26.50	0.09
C	15.90	−573.74	351.28	−2035.28	−108.54
pHxT	0.01	−0.04	0.01	1.64	−0.02
pHxC	1.31	60.69	−29.44	−1772.78	16.54
TxC	−1.19	−0.18	−2.44	155.83	0.31

T, turbidity; S, sedimentation; ζP, ζ potential; PS, particle size; CI, color index.

**Table 7:** Optimal values of the properties of the NSH suspension.

Condition Criteria	Parameters	Minimum *	Maximum *	Optimum	Case Application for Day 20
Restriction	pH	4.00	6.50	4.50	4.50
	T (°C)	40.00	90.00	84.55	85.00
	C (g/L)	0.05	0.15	0.08	0.08
Objective function (maximize)	Sedimentation (%T)	---	---	72.34	74.33 ± 0.35
Subject to	Turbidity (%T)	90.00	99.00	99.00	99.27 ± 0.83
	ζ potential (mV)	---	---	−25.64	−26.55
	Particle size (nm)	300.00	400.00	300.00	311.21 ± 21.54
	Color index	−2.00	2.00	−2.00	−2.14 ± 0.09

* Declared values considering extrapolation in the LITF model (linear with interaction of two factors).

## Data Availability

Data are contained within the article.
